# Atomic Force Microscopy and Molecular Dynamic Simulation of Adsorption of Polyacrylamide with Different Chemistries onto Calcium Carbonate

**DOI:** 10.3390/polym16040494

**Published:** 2024-02-10

**Authors:** Jin Hau Lew, Keat Yung Hue, Omar K. Matar, Erich A. Müller, Paul F. Luckham, Adrielle Sousa Santos, Maung Maung Myo Thant

**Affiliations:** 1 Department of Chemical Engineering, Imperial College London, London SW7 2AZ, UK; v.hue20@imperial.ac.uk (K.Y.H.); o.matar@imperial.ac.uk (O.K.M.); e.muller@imperial.ac.uk (E.A.M.); p.luckham01@imperial.ac.uk (P.F.L.); adryellesousasantos@gmail.com (A.S.S.); 2 PETRONAS Research Sdn. Bhd., Lot 3288 & 3289, Off Jalan Ayer Itam, Kawasan Institusi Bangi, Kajang 43000, Selangor, Malaysia; maungmyothant@petronas.com

**Keywords:** atomic force microscopy (AFM), force spectroscopy, molecular dynamic (MD) simulation, hydrolysed polyacrylamide, sulfonated polyacrylamide, adsorption, salt

## Abstract

This study investigates the interaction of polyacrylamide (PAM) of different functional groups (sulfonate vs. carboxylate) and charge density (30% hydrolysed vs. 10% hydrolysed) with calcium carbonate (CaCO_3_) via atomic force microscopy (AFM) and partly via molecular dynamic (MD) simulations. The PAM used were F3330 (30% hydrolysed), AN125 (25% sulfonated), and AN910 (% hydrolysed). A total of 100 ppm of PAMs was prepared in 0.1% NaCl, 3% NaCl, and 4.36% NaNO_3_ to be employed in AFM experiments, while oligomeric models (30 repeating units) of hydrolysed polyacrylamide (HPAM), sulfonated polyacrylamide (SPAM), and neutral PAM (NPAM) were studied on a model calcite surface on MD simulations. AFM analysis indicated that F3330 has a higher average adhesion and interaction energy with CaCO_3_ than AN125 due to the bulky sulfonate side group of AN125 interfering with SPAM adsorption. Steric repulsion of both PAMs was similar due to their comparable molecular weights and densities of the charged group. In contrast, AN910 showed lower average adhesion and interaction energy, along with slightly longer steric repulsion with calcite than F3330, suggesting AN910 adopts more loops and tails than the slightly flatter F3330 configuration. An increase in salt concentration from 0.1% to 3% NaCl saw a reduction in adhesion and interaction energy for F3330 and AN125 due to charge screening, while AN910 saw an increase, and these values increased further at 4.36% NaNO_3_. MD simulations revealed that the salt ions in the system formed salt bridges between PAM and calcite, indicating that the adhesion and interaction energy observed from AFM are likely to be the net balance between PAM charged group screening and salt bridging by the salt ions present. Salt ions with larger bare radii and smaller hydrated radii were shown to form stronger salt bridges.

## 1. Introduction

Carbonate reservoirs are invaluable natural gas reserves [1,2], yet many are often poorly consolidated with weak and loosely packed structures due to their young geological age [3,4]. Any increase in the effective stress is very likely to cause irreversible deformation on these weak formation rocks [5,6,7], leading to fines migration that decreases hydrocarbon yield due to pore clogging, failures in processing equipment, and increased operational costs associated with the disposal of fines [8]. Various strategies have been developed to mitigate this issue, falling into two main categories: mechanical methods and chemical methods. Among these, chemical methods have gained significant attention due to their advantages over mechanical techniques, as chemical methods involve the injection of formation-strengthening chemicals while avoiding mechanical complexities, including installation difficulties, maintenance challenges, and potential damage to the wellbore [9,10,11,12].

**Figure 1:**
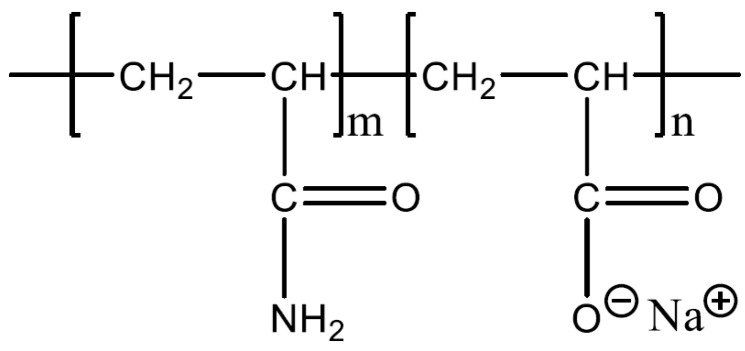
Chemical formula of HPAM [23].

Polyacrylamide (PAM), commonly used in enhanced oil recovery (EOR) [13,14], receives attention as a potential consolidation candidate. While many studies have been conducted on applying PAMs onto sandstone, the application of PAMs onto carbonate rocks requires further study. Apart from the compositional difference, the natural charge of sandstones and carbonate rocks in reservoir conditions differs. A useful parameter to determine the natural charge is the isoelectric point (IEP), which gives the point of zero zeta potential on the surface [15]. If the surrounding pH is lower than the pH_IEP_, the substrate will possess a positive surface charge, and vice versa [16,17,18]. Most reported pH_IEP_ of carbonate rocks are usually between pH 8 and 11 [19,20,21], while the pH of the formation water usually ranges between 4 and 9 [22]; thus, carbonate rock in the reservoir usually exhibits a positive surface charge. Thus, a negatively charged polymer such as hydrolysed polyacrylamide (HPAM) is a logical candidate to consolidate loose carbonate rock particles. The hydrolysation of acrylate monomer into a carboxylate group (COO^−^) gives rise to the negative charge of this polymer, as shown in Figure 1 [23].

**Figure 2:**
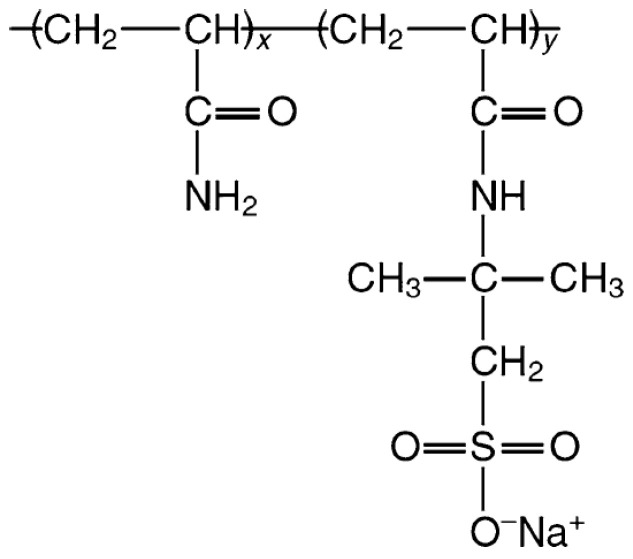
Structure of sulfonated polyacrylamide (SPAM) [29].

The degree of hydrolysation of PAM affects the resulting charge density HPAM and, thus, the adsorption kinetics and polymer configuration on the surface. One such study was conducted by Rasteiro et al. [24], which involved examining the effect of cationic polyacrylamide (CPAM) charge density on its adsorption onto negatively charged precipitated calcium carbonate. The lower charged CPAM showed lower adsorption equilibrium and slower adsorption kinetics than higher charged CPAM. In the liquid phase, highly charged HPAM undergoes a chain expansion due to the repulsion of similarly charged carboxylate (COO^−^) segments. At the same time, weakly charged HPAMs will adopt a more coiled conformation with random walk characterisation [25]. When the polymer is on the surface, weakly charged HPAMs tend to form shorter train segments with the surface, thus adopting more loops and tails, favouring bridging interactions. This is contrary to the behaviour exhibited by highly charged HPAMs, favouring patch attraction and adopting a flatter configuration on a surface [8,26,27,28]. The functional group of polyacrylamides (PAMs) can also be altered to give different properties to the polymer. One such group is the copolymer of acrylamide and acrylamide tert-butyl sulfonate (ATBS), which is also known as sulfonated polyacrylamide (SPAM) [29]. The chemical formula of SPAM is shown in Figure 2.

The difference between SPAM and HPAM is that the carboxylate functional group of HPAM is replaced by a sulfonate functional group (SO_3_^−^), as shown in Figure 2. The reason several authors considered the usage of SPAM instead of HPAM is the apparent superior thermal and brine stability of SPAM than HPAM. Rashidi et al. [30,31] compared the viscosity and retention of SPAM and HPAM in porous sandstone under high salinity and temperature conditions. SPAM exhibited a smaller viscosity reduction compared to HPAM of similar molecular weight in synthetic seawater up to 80 °C. Rashidi et al. [30,31] proposed that the ATBS functional group of SPAM provided an increased thermal stability to the polymer molecular structure. Sorbie [32] observed that the retention of SPAM within sandstone is significantly lower than that of HPAM, with a further decrease in retention with increasing degree of sulfonation. Thomas et al. [33] attributed the lower adsorption affinity of SPAM as compared to HPAM to the sulfonate side groups, which are bulkier than the carboxylate groups, hence interfering with surface adsorption. However, Salehi et al. [34] observed a different result when SPAM crosslinked with chromium triacetate was subjected to high salinity and temperature. They showed that the critical strain where SPAM lost its viscoelastic property decreased in salt solution, and this deterioration is more severe in divalent than in monovalent salt (−200% in NaCl vs. −500% in CaCl_2_). The reason Salehi et al. [34] proposed was that the salt cations compete and disrupt the chromium (III) ions to bond with the sulfonate functional group, causing a reduction in viscosity.

Sodeifian et al. [35] also studied the adsorption of SPAM onto calcium carbonate rock and attributed the adsorption of negatively charged SPAM onto the positively charged carbonate rock to attractive electrostatic interactions. They also observed that when the salinity of the polymer solution increased, there was a decrease in the adsorption of SPAM onto the CaCO_3_ particles due to the screening of the negatively charged sulfonated groups of SPAM chain by the salt cations, which caused a decrease in the electrostatic interaction between SPAM and CaCO_3_. Several authors also mentioned that the contribution of salt ions toward polymer adsorption onto a surface depends on the nature of the electrostatic forces between the two molecules. That is, if the original electrostatic interaction between the adsorbate and adsorbent is attractive, an introduction of salt will decrease the adsorption process, and vice versa [36,37,38,39].

The interaction of rock surfaces with anionic polyacrylamides of different functional groups (sulfonate vs. carboxylate) and charge densities, in the presence of salinity-related effects, can be understood through atomic force microscopy (AFM). This topographical imaging technique involves raster-scanning the surface of a sample using a sharp tip to detect unique surface features and converting them into images along with their force profiles. One such work was performed by Al-Hashmi et al. [13], where the authors conducted the adsorption of non-ionic polyacrylamide (NPAM), hydrolysed polyacrylamide (HPAM), and sulfonated polyacrylamide (SPAM) on a glass surface. In this work, both AFM and quartz crystal microbalance with dissipation monitoring (QCM-D) were employed. In the QCM-D result, they observed the adsorbed number of polyacrylamides onto glass surface followed the sequence of NPAM > HPAM > SPAM. The fact that HPAM displayed lower adsorption onto glass than NPAM was simply due to the repulsion of the negatively charged glass surface by the anionic carboxylate group on HPAM. Comparing HPAM and SPAM, the lower adsorption of SPAM was attributed to the non-bonding and bulky ATBS group. This trend was also reflected in the AFM force spectroscopy analysis, where at 20 min of glass incubation in polyacrylamides, the adhesion of the retraction curve was the largest in NPAM, followed by HPAM and SPAM, further corroborating their hypothesis. 

On the other hand, molecular simulation of polymers adsorption onto mineral surfaces have gained increasing interest to complement experimental work in screening potential polymer candidates, allowing better understanding of the polymers–mineral interaction. Molecular dynamic (MD) simulation is one such study to investigate polyacrylamide adsorption onto calcite surfaces while providing insight into the contribution of functional groups to the adsorption mechanism. Research involving MD simulation of polymers adsorption onto mineral surface [40,41,42] has been reported, including the parametric studies of the polymers functional group [43,44], charge density [45,46,47], and salinity effect [48,49,50]. One such piece of literature closely related to our study, despite different mineral surfaces, is from Quezada et al. [51,52,53,54] who simulated the adsorption of anionic polyacrylamide (APAM) onto a quartz surface with different surface charge densities and salt ions at varying pH conditions. As quartz consists of negatively charged silica, the salt cations act as salt bridges to facilitate the adsorption of anionic APAM to the surface, while the polymer adsorption is hindered by increasing surface charge density and higher pH level due to the increasing negative repulsion force between the polymer and the mineral surface.

Despite these interesting findings, the parametric study of anionic polyacrylamide (APAM) interaction with calcium carbonate (CaCO_3_) surfaces is very scarce. It is useful to understand the underlying interaction to better facilitate any effort of CaCO_3_ consolidation by polyacrylamide (PAM). Thus, AFM experiments were conducted to study the effect of the PAM functional group, charge density, and the effect of salt concentration on their interaction with calcium carbonate. At low HPAM charge density, experiments were also implemented to investigate the effect of salt anions on possible salt bridging. This work is a continuation of our previous work [55], but the novelty of this work is the comparison on the adsorption of PAM of sulfonated functional group and carboxyl functional group and the adsorption of PAM having two different densities of charged carboxyl functional group through a force mapping study to compile primarily the adhesions and interaction energies over a particular area. MD simulations were also conducted to provide better insight into the interaction of these PAMs with different chemistries with the CaCO_3_ surface.

## 2. Materials and Methods

### 2.1. Materials

Iceland Spar calcite crystals, which are rhombohedral and colourless, were purchased from Manchester Minerals (Shrewsbury, UK). Three types of anionic polyacrylamides (APAMs) were provided by SNF Floerger (Wakefield, UK), namely F3330S (30% hydrolysed, 11–13 MDa), AN910 (10% hydrolysed, 11–13 MDa), and AN125 (25% sulfonated, 8 MDa). The calcium carbonate powder (CaCO_3_, ≥99%) and sodium chloride (NaCl, 99.5%) were purchased from VWR Chemicals Ltd. (Lutterworth, UK), while sodium nitrate (NaNO_3_, extra pure) was purchased from Thermo Fisher Scientific (Dartford, UK). Solutions used in this work were prepared using deionised water (DI, 18 MΩ). The AFM experiments were conducted under room conditions (25 °C, 1 atm).

### 2.2. Experimental Procedures

#### 2.2.1. Preparation of Polymer Solutions

The solvent used in this work was NaCl solution of 0.1 and 3 wt% and NaNO_3_ of 4.36 wt%. A total of 0.1 wt% NaCl was prepared to remove any electrostatic interaction between the bare surfaces, which might interfere with the interpretation of the adsorbed polymer layers [56], while the 3 wt% NaCl was prepared to imitate naturally occurring reservoir conditions. In the case of AN910, we also prepared 4.36 wt% NaNO_3_ to compare the result with that using 3 wt% NaCl to study the effect of salt anion on HPAM interaction with CaCO_3_. A total of 4.36 wt% NaNO_3_ had a similar ionic strength to 3 wt% NaCl. The polymer solutions were prepared according to API-RP-63, replacing DI water with brine. Here, a mother stock of 15,000 ppm was prepared by adding the polymer powders on the shoulder of the vortex of fast-stirring DI water to prevent the formation of fisheye polymer agglomerates. After stirring for more than 24 h, the homogenous mother stock was diluted into 100 ppm of stock solution using the salt solutions prepared.

#### 2.2.2. Calcite Crystal Incubation with Polyacrylamides

Iceland Spar crystal was cleaved into smaller pieces with dimensions of approximately 10 mm × 10 mm × 3 mm for nanoscale solid–liquid interfacial experiments. Cleaving was performed under room condition using a clean bladed tool to expose the smooth original surface of the crystal. The cleaved samples were immediately cleaned with DI water and absolute ethanol (Sigma Aldrich, ≥99.5%, Merck Life Science UK Limited, Dorset, UK) to remove possible dust contaminants from the surface. Once the liquid dried off, the cleaved samples were placed in a clean Petri dish where the polyacrylamide samples were added dropwise into the dish until the crystals were fully covered with the polymers. The Petri dish was immediately sealed to prevent dust intrusion and were left to be incubated for more than 18 h, which was the minimum time for optimum adsorption from our previous work [27].

#### 2.2.3. Atomic Force Microscopy (AFM)

All AFM experiments in this work were conducted using a JPK NanoWizard 4 atomic force microscope, and the procedure was identical from our previous work [55]. A NanoWorld^®^ Pyrex-Nitride Probe—Triangular AFM Cantilevers (PNPTR) (cantilever length: 100 µm, tip height: 3.5 µm, tip radius: <10 nm, nominal force constant: 0.32 N/m) was used in every AFM scan. Calcium carbonate particles (CaCO_3_, ≥99%, VWR Chemicals Ltd.) with average diameter of 3.5 µm were attached onto the tip of the AFM cantilever under a microscope using a micromanipulator (Melles Griot, Rochester, NY, USA) to ensure the interaction registered by the AFM was always between polyacrylamides (PAMs) and calcium carbonate (CaCO_3_). All measurements were performed in the liquid mode, and the calibration of the cantilever was performed through the instrument’s slope correction and thermal noise function, which gave an experimental cantilever stiffness of 0.3 N/m. In this study, the samples were scanned principally with the force mapping (FM) mode. The AFM tip measured the topography of the surface pixel-by-pixel along a fast and slow axis, which produced a force profile for parameters adhesion and interaction energy [57]. These parameters were essential points of comparison with the MD simulation results. 

For the imaging of the calcite surface, the scan size was typically between 0.5 and 1 µm^2^ with 16 × 16 pixels, each pixel having a contact time of 2 s and approach velocity of 2 µm/s. The setpoint and the z-length of this work were 0.5 nN and 300–500 nm, respectively. A minimum of four force map area scans were conducted, and the experiments were duplicated, in which a new AFM cantilever, attached with fresh CaCO_3_, would be used in every new experiment to prevent the saturation of CaCO_3_ particles by possible polyacrylamide molecules from previous scans. A minimum of 1000 force scans were produced, and the net interaction of CaCO_3_ with PAM was observed as the system reached equilibrium.

#### 2.2.4. MD Simulation Methods

Simulation system preparation. Classical atomistic MD simulations were performed to simulate the polymer adsorption onto carbonate surfaces. Material Exploration and Design Analysis (MedeA) simulation software version 3.5 [58] using the computation engine LAMMPS [59] was employed. Calcite structure was modelled to represent the carbonate rock. The unit cell was a rhombohedral crystal structure with space group of *R*$\bar{3}$c, where it had a dimension of *a* = *b* = 4.980 Å, *c* = 17.192 Å, and a plane angle of *α* = *β* = 90°, *γ* = 120°. It was further cleaved into crystal plane (1 0 4), as it was the most thermodynamically stable structure and had lowest surface free energy as validated in our previous work [60]. The crystal plane was replicated as 6-layer thickness structure with 720 CaCO_3_ molecules and dimensions of *a* = 48.86 Å, *b* = 49.76 Å, and *c* = 17.84 Å. A total of 3000 water molecules were placed above the calcite structure with a single polymer chain placed within the water solvent. The polymer structures were created to mimic the experimental candidates. To consider the effect of the polymer functional group in a saline environment, three polymer candidates, each with 30 repeat units, were modelled, including neutral PAM (NPAM) (the repeat unit consisted of 3 acrylamide monomers), HPAM with charge density 33% (the repeat unit consisted of 2 acrylamide monomers and 1 acrylate monomer), and SPAM with charge density 33% (the repeat unit consisted of 2 acrylamide monomers and 1 acrylamide tert-butyl sulfonate or ATBS monomer). NPAM, HPAM 33%, and SPAM 33% were surrogates of AN910, F3330, and AN125, respectively. The charged polymers, HPAM 33% and SPAM 33%, were modelled with the same charge density to ensure consistent comparison, and 30 Na ions were placed to their respective system to ensure electroneutrality in the simulation environment. A total of 29 NaCl molecules were placed in the water for all polymer systems to create the salinity of 3 wt%. A layer of vacuum region was added in the simulation box to isolate one of the calcite surfaces and direct the adsorption of polymer onto a single calcite surface layer. The resulting simulation box had dimensions of *a* = 48.86 Å, *b* = 49.76 Å, and *c* = 86.38 Å. To investigate the effects of anions on NPAM, the simulations were repeated by replacing NaCl molecules with NaNO_3_ molecules. Simulation details are explained in the supporting information.

## 3. Results and Discussion

### 3.1. Effect of Functional Group

**Figure 3:**
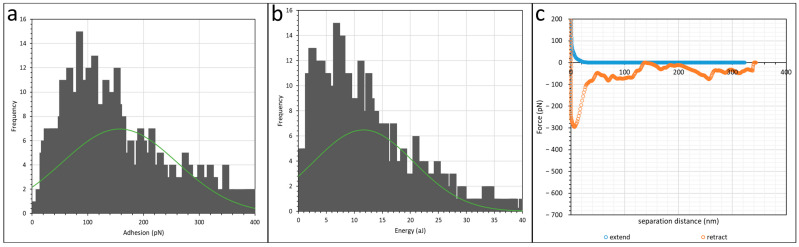
Histogram plot of (**a**) adhesion; (**b**) interaction energy of calcite immersed in AN125 in 0.1% NaCl. Green curve shows normal distribution plot; (**c**) most recurring retract force curve shape at AN125 in 0.1% NaCl.

The effect of polyacrylamide functional groups on its interaction with calcium carbonate was investigated by comparing sulfonated polyacrylamide (SPAM) and hydrolysed polyacrylamide (HPAM), namely AN125 (25% sulfonated) and F3330 (30% hydrolysed), respectively. These two polymers have very similar molecular weights and percentages of charged groups. Here, two cleaved Iceland Spar calcite crystals were incubated separately in both polyacrylamides (PAMs) with a concentration of 100 ppm dissolved in 0.1 wt% NaCl. Four scan areas of 16 × 16 pixels were scanned under force mapping, and the experiments were repeated twice. The adhesion value (the largest peak of the retract force curves) and the interaction energies (the area under the curves) were compiled into histograms, with their respective green normal distribution plot. The average and peak values from scans on AN125 are tabulated in Table 1, with its histogram plots and respective green normal distribution plots displayed in Figure 3. The most recurring force–distance curves for SPAM interaction are also displayed in Figure 3c. Note that only one set of histograms is presented due to their qualitative similarities. The magnitude of adhesion and interaction energy can be observed from the horizontal axis in Figure 3a,b. The force mapping results for F3330 (HPAM) can be read from our previous publication [55]. As a quick summary, the force mapping results of F3330 on CaCO_3_ are as followed: steric repulsion distance = 30–70 nm; average adhesion = 450–625 pN; peak adhesion = 266–566 pN; average interaction energy = 43–145 aJ; and peak interaction energy = 29–66 aJ. 

The absolute magnitude of adhesion and interaction energy in these AFM scans were usually difficult to reproduce, as shown in Table 1. This could be due to different reasons such as different number of PAMs adhering to CaCO_3_ on the AFM tip, the configuration of each polymer molecule on the surface, and the possibility of residual PAM molecules from previous scans adhering to the CaCO_3_ particles on the AFM tip. However, the trend obtained from each experiment was reproducible, which was the desired outcome. From Table 1, it is observed that the adhesion and interaction energies of F3330 with CaCO_3_ [55] were typically larger than that of AN125 with CaCO_3_. This was an expected result, since SPAM possessed sulfonate side groups, which are bulkier than the carboxylate groups, hence interfering with the surface adsorption [33]. Similar findings were reported by Plank and Sachsenhauser [61], where they mentioned that the lower adsorption of polymers with larger side chains is due to higher surface occupancy of the larger side group. The range of steric repulsion distance of both F3330 and AN125 detected by the AFM was very similar. This was probably due to both polymers having very similar molecular weights and densities of charged groups in their polymeric chains. 

**Figure 4:**
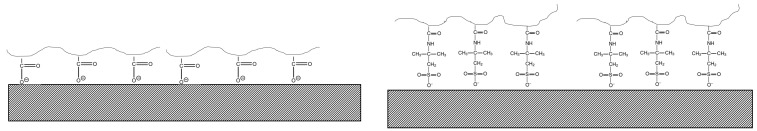
Proposed HPAM (**left**) and SPAM (**right**) configurations on calcite crystal surface.

Referring to Figure 3c, the retraction force curves of F3330 [55] and AN125 are qualitatively similar, which usually possess an initial peak followed by either a flat or zig-zagged plateau region. The proposed explanation of this phenomenon was illustrated in our previous publication [55], where the simultaneous detachment of large amounts of anchor points produced a sharp sudden drop in adhesion, with the only difference between the two being the magnitude of the initial peak being smaller in SPAM than HPAM. This suggests that the configuration of both PAMs on the calcite surface is very similar, which likely adopts a train-like conformation with the charged functional group of the PAMs forming multiple anchor points on the surface [62,63]. Combining the explanations above, the configuration of F3330 and AN125 on the calcite surface is proposed as in Figure 4, where the sulfonated group of SPAM clearly posed greater hinderance for effective adsorption onto calcite surface than carboxylate group of HPAM.

### 3.2. Effect of Charge Density

**Figure 5:**
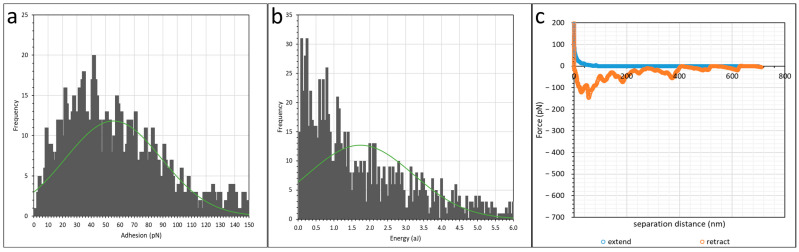
Histogram plot of (**a**) adhesion; (**b**) interaction energy of calcite immersed in AN910 in 0.1% NaCl. Green curve shows normal distribution plot; (**c**) most recurring retract force curve shape at AN910 in 0.1% NaCl.

The effect of polyacrylamide charge density on its interaction with calcium carbonate was investigated by comparing between two HPAMs of similar molecular weights, namely F3330 (30% hydrolysed) and AN910 (10% hydrolysed). The AFM scan procedure was similar as in the previous section. The average and peak values of adhesion and interaction energy from scans on AN910 are tabulated in Table 2. Histogram and green normal distribution plots along the most recurring force–distance curve for the interaction of AN910 are displayed in Figure 5. The force mapping results of F3330 were mentioned in Section 3.1. 

The interaction energy histogram plot in Figure 5 is skewed rather to the left; thus, it is helpful to record the peak value from the histogram. From Table 2, it is observed that the adhesion and interaction energy values of AN910 were significantly lower than that of F3330. This is expected as lower charged AN910 has lesser charged carboxylate (COO^−^) groups which could interact electrostatically with the positively charged surface than F3330. Thus, when AN910 is on calcite surface, presumably, it forms fewer train segments with the surface and adopts more loops and tails configurations, which is contrary to F3330, which is more likely to adopt a flatter configuration on a surface [26]. This also explains the slightly longer steric repulsion distance in AN910 than in F3330 (40–90 nm vs. 30–70 nm). A similar finding was discussed by Zhou et al. [64] where they also observed significantly larger adhesion upon retraction when a higher charge density polymer was involved. It is interesting to observe that the values in AN910 were not approximately three times lower than F3330, given the fact that the reported charge density by the manufacturer of AN910 is three times lower than F3330. Hence, this suggests that while the adhesion and interaction do increase with increasing charge density, it does not necessary follow that it increases proportionally with charge density. The dominant shape of retraction force–distance curve in Figure 5c is mainly made up of small zig-zag peaks, which is due to the sequential detachment of weakly adhering carboxylate (COO^−^) group from the calcite surface [63]. An illustration of the polymer detachment sequence of this sort of force curve is similar to that of F3530 in our previous work [55].

### 3.3. Effect of Salt

The effect of salinity on the three PAMs experimented above was studied by replacing the salt from 0.1% NaCl to 3% NaCl, in which the ionic strength is close in value to that of a naturally occurring reservoir brine. The average and peak adhesion and interaction energy for two batches of experiments for AN125 and AN910 are tabulated in Table 3. The histograms and the recurring force curves were omitted as they do not convey any new information, which is not obtainable from the values in Table 3. The force mapping results of F3330 in 3% NaCl can be obtained from our previous publication [55]. Again, as a quick summary, the force mapping results of F3330 in 3% NaCl are as followed: steric repulsion distance = 60–120 nm; average adhesion = 72–120 pN; peak adhesion = 32–131 pN; average interaction energy = 5.6–17 aJ; and peak interaction energy = 2.9–23 aJ.

The batch number in Table 1, Table 2 and Table 3 indicates that the experiments were conducted with the same setup. For example, the AFM tip and crystal of AN125 in batch 1 is the same in both 0.1% and 3% NaCl, thus direct comparison between low and high salt can be made between experiments of the same batch number, likewise, for the other two polymers. Table 3 shows that the adhesion and interaction energy values for F3330 [55] and AN125 decrease with increasing salt concentration, and this is particularly noticeable for the peak force. This is an expected result, as the Na^+^ ions from the salt are expected to screen the negatively charged segment of F3330 and AN125, thus reducing the electrostatic attraction between the polymers and the positively charged calcite surface [29,65]. With this reduction in electrostatic attraction, the PAM molecules are more loosely bound to the calcite surface, thus extending further to be detected by the AFM tip in the form of increased steric repulsion distance. However, this explanation becomes invalid when considering the increase in adhesion and interaction energy of AN910 from 0.1% to 3% NaCl. Thus, it is possible that at high salt concentration, charge screening might not be the only mechanism at work. Consequently, attention was given to molecular dynamic (MD) simulation for any possible alternative in explanation. 

**Figure 6:**
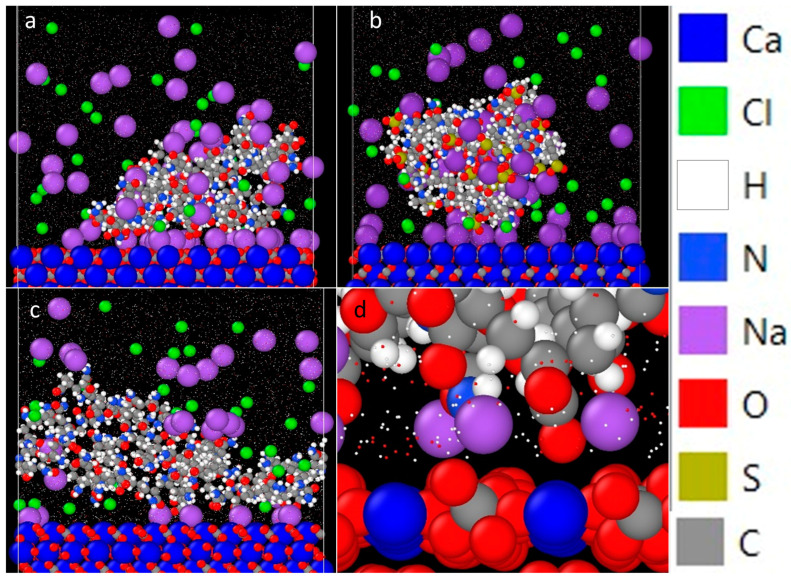
Snapshot of the adsorption equilibrium of (**a**) HPAM 33%, (**b**) SPAM 33%, and (**c**) NPAM onto calcite in the presence of NaCl; and (**d**) detail of the positioning of Na ions (purple) between O group (red) of polyacrylamides and calcite surface. Legend for colour-coding the atoms in MD simulation is displayed on the right.

Results from MD simulations of HPAM 33%, SPAM 33%, and NPAM adsorption onto a calcite surface at equilibrium are shown in Figure 6, along with the position of Na^+^ ions between the O group of the polyacrylamides and calcite surface. All these figures share the same colour code. Water molecules were shrunk dramatically to clearly display the interaction between polymer and the surface in the presence of salt, and the sodium atom was enlarged to display the effect of salt bridging. A reminder that the polymer molecules in the MD simulations are meant to be an analogue of actual polymer used in the experiment, and they do not reflect the actual polymers. This is because the polymers used in the experiments are very large in molecular weight, which is impossible to be simulated in MD. Behaviour of F3330 on calcite is represented by HPAM 33%, AN125 by SPAM 33%, and AN910 by NPAM.

Results from the MD simulation suggest that the Na^+^ ions tend to orient themselves between the polymer and the calcite surface, which is a manifestation of salt bridging by salt ions between the two surfaces. The phenomenon of salt bridging here is rather surprising, as the expectation is that in an oppositely charged adsorbent and adsorbate pair, the salt ions reduce the adsorption process by screening the charged groups. It is only when both surfaces are repulsive that the salt ion facilitates adsorption in the form of salt bridging [35,36,37,38,39]. By observing Figure 6d, it is observed that the Na^+^ ions are found between the O groups belonging to the PAMs and CO_3_ on the calcite surface. The fact that the Na^+^ ions always favour approaching the O group of the PAMs is consistent with charge screening but yet these ions, apparently, still play a role in bridging between the polymer and the surface. The result from the MD simulations here supports the inference from AFM results. Our MD and AFM results suggest, collectively, that the effect of salt ions in the consolidation of carbonate rock is a balance between charged group screening and salt bridging. A similar phenomenon was observed in the work of Pérez et al. [66], whereby they proposed the effectiveness of flocculation of clay by cationic polyacrylamide is a combination of salt bridging (which facilitates higher flocculation) and electrostatic shielding of polymer functional group (which hinders flocculation). 

By comparing the MD simulation results for HPAM 33% and SPAM 33% in Figure 6, respectively, it is clearly seen that the degree of salt bridging is higher in the former case since HPAM 33% molecules adhere closer to the surface than their SPAM 33% counterparts. This is due to the bulkier sulfonated group of SPAM, which led to higher steric repulsion for effective salt bridging. The net decrease in adhesion and interaction energy values in HPAM 33% and SPAM 33% is probably due to the electrostatic attraction directly between PAM charged group and the calcite surface having been replaced by a salt bridging (an intermediary salt cation), in which, here, there is no direct contact between PAM and the calcite surface. Comparing HPAM 33% and NPAM from Figure 6, the degree of bridging by Na^+^ ions in NPAM is lower than in HPAM 33% simply due to NPAM having little-to-no charged COO^−^ group available for bridging. However, the Cl^-^ ions seemed to gather between the NPAM and calcite surface; thus, Cl^-^ might be another salt bridging ion in play here. 

**Figure 7:**
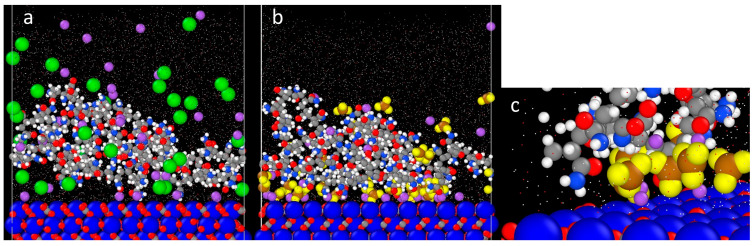
Snapshots of NPAM adsorption onto calcite in the presence of (**a**) NaCl (green particles are the Cl^−^ ions and (**b**) NaNO_3_ (yellow and brown particles are the NO_3_^−^ ions); (**c**) detail of the orientation of NO_3_^−^ ions (yellow–brown atoms) between NPAM and calcite surface. NO_3_^−^ ions have been enlarged to emphasise their orientation. Other atoms follow same colour scheme as Figure 6.

**Figure 8:**
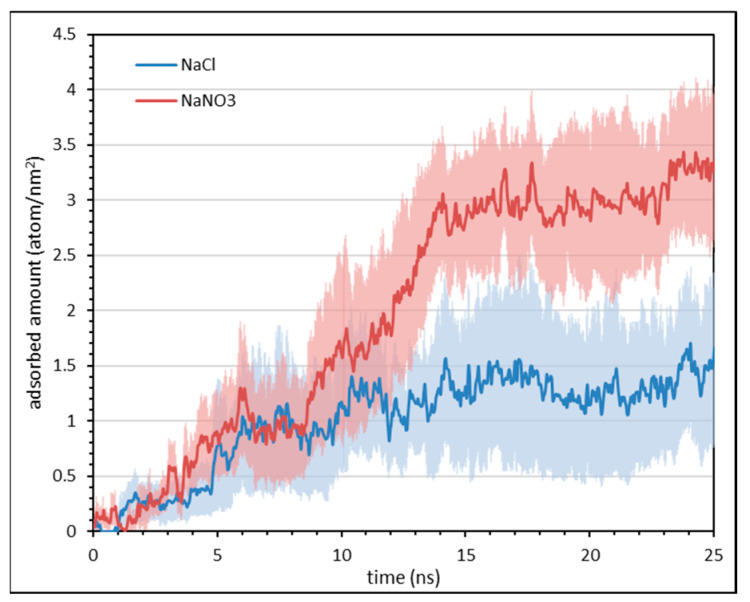
Simulation time profile of adsorbed amount of NPAM onto calcite surface in different salt from MD simulation. The average adsorbed amount is taken from last 5 ns of data. Translucent light colour area around the darker colour curves represents the standard error of each curve from 5 different realisations.

**Figure 9:**
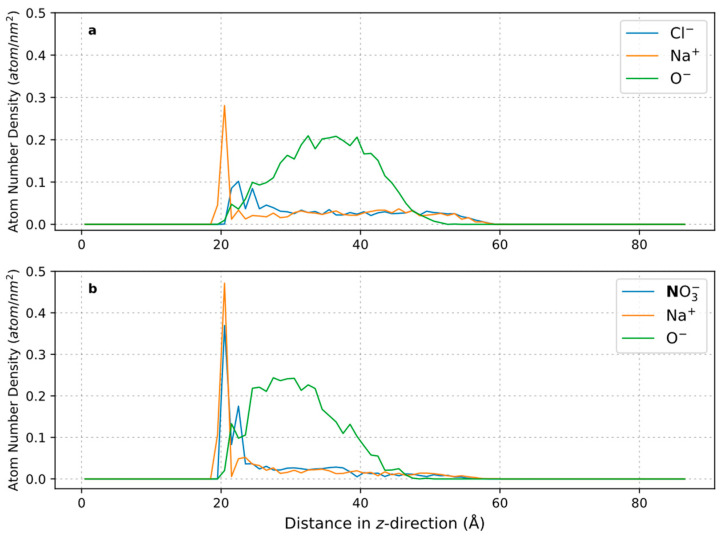
Polymer adsorption number density profile on calcite surface with distance distribution along z-direction, averaged over last 5 ns of production and run from 5 different realisations. (**a**) NaCl adsorption. Blue: chloride ion as salt anion, orange: sodium ion as salt cation, and green: oxygen atom from polymer carbonyl group (C=ONH_2_); (**b**) NaNO_3_ adsorption. Blue: nitrogen atom from nitrate ion as salt anion, orange: sodium ion as salt cation, and green: oxygen atom from polymer carbonyl group.

To further elucidate the phenomenon of salt bridging, the work of Al-Hashmi et al. [67] on the effect of salt ion bare and hydrated radii on the facilitation of salt bridging between polymer and surface was referred. In their work, they discovered that the amount of salt bridging increased when the salt cations’ bare radii increased and hydrated radii decreased. Thus, 3% NaCl was replaced with equimolar of NaNO_3_ (4.36 wt%), where the hypothesis is that the NO_3_^−^ should be a more effective salt bridging ion than Cl^−^, since NO_3_^−^ has a larger bare radius and a smaller hydrated radius than Cl^−^. Both AFM force mapping and MD simulations were conducted to test this hypothesis, and the results are compiled below. The compilation of histogram values from AFM force mapping in Table 4 shows that the average and peak adhesion and interaction energy values of AN910 with calcite under NaNO_3_ are higher than that in NaCl. For MD simulation results presented in Figure 7, the polymers were allowed to equilibrate for 30 ns, where the adsorbed amount is taken as the number of atoms on the polymer molecule adsorbed on the surface (1 nm^2^) with the average of the data points from the last 5 ns. These results demonstrate that the NPAM molecules are able to approach closer to the calcite surface in the presence of NO_3_^−^, which are the brown (N) and yellow (O) ions in Figure 7, than in Cl^−^, represented as green ions. This is further corroborated by the results shown in Figure 8, in which the MD simulations indicated that the adsorbed amount of NPAM onto calcite surface in NaNO_3_ at equilibrium is higher than in NaCl, with values of approximately 3 atoms/nm^2^ ± 0.67 and 1.5 atoms/nm^2^ ± 0.62, respectively, for NaNO_3_ and NaCl. A density distribution analysis of the atom type representing the salt cation, anion, and polymer for their adsorption on the calcite surface was further evaluated in Figure 9, where the calcite surface is located at a distance of approximately 18 Å.

Based on the results presented in the foregoing, the following explanation is proposed: AN910 has the lowest initial charge density of 10% among the experimented anionic PAMs, thus very little direct electrostatic attraction between the charged carboxylate (COO^−^) group and positively charged calcite surface is anticipated. It is possible that the charge screening of salt ions essentially rendered the AN910 a non-ionic polyacrylamide (NPAM). Since the reported IEP of CaCO_3_ is typically around 9–10 [22], the calcite surface in our work is positively charged under NaCl solution of around pH 7. Al-Hashmi et al. [67] reported that NPAM relies principally on the partial positive hydrogen atom of NH_2_ to perform hydrogen bonding with a surface. In this case, since both hydrogen atom and calcite surface are positive in nature, the most logical salt ion involved in salt bridging would be the salt anion. Therefore, the increase in adhesion and interaction energy of AN910 at 3% NaCl is very likely to be contributed by the salt bridging of Cl^-^ between the hydrogen atom of amide group and the calcite surface. When 3% NaCl is replaced by equimolar NaNO_3_, the adhesion and interaction energy further increased, which is in line with the theory put forth by Al-Hashmi et al.’s work, [67] stating that the NO_3_^−^ ion has a larger bare radius and smaller hydrated radius than Cl^−^, making it a more effective bridging ion. By observing the orientation of NO_3_^−^ ions between NPAM and the calcite surface from MD simulation in Figure 7c, the NO_3_^−^ ions were oriented by having their O atoms (yellow) facing the H atoms (white) of the polyacrylamide and the Ca atoms (blue) of the calcite surface, further corroborating the proposed hypothesis. This result is further verified from the polymer density profile from MD in Figure 9. First, it is observed that in both plots, there is a significantly high adsorption density of the salt ions on the calcite surface, which indicates the role of salt bridging to facilitate the adsorption of polymer on the calcite. From Figure 9b, the green plot shows that the peak of the polymer adsorption density is shifted a relatively closer distance to the calcite surface. This again shows that salt bridging is more effective in nitrate ion due to the higher adsorption surface density near the calcite surface, which enables the polymer to be adsorbed in closer proximity to the calcite surface than chloride ion.

## 4. Conclusions

Atomic force microscopy (AFM) of Iceland Spar calcite crystal incubated in anionic polyacrylamides (PAMs) with different functional groups and charge densities was conducted. These PAMs were prepared in different salt solutions at 0.1% NaCl, 3% NaCl, and 4.36% NaNO_3_. MD simulations on model calcite surfaces were performed for oligomeric models of the polymers. AFM analysis of F3330 and AN125 showed that while both have a similar steric repulsion range, a higher adhesion and interaction energy are observed in hydrolysed polyacrylamide (HPAM) compared to sulfonated polyacrylamide (SPAM) due to bulkier sulfonate side groups of SPAM interfering with adsorption onto the calcite surface [33]. On the other hand, AN910 exhibited lower adhesion and interaction energy than F3330 due to its lower charge density and, hence, interacts less with the positively charged calcite. The steric repulsion observed at AN910 is also slightly longer ranged than F3330, suggesting that the AN910 molecules form fewer train segments with the surface and adopt more loops and tails configurations, in contrast to a flatter F3330 configuration on the surface [26]. Increasing salt concentration from 0.1% to 3% NaCl decreased adhesion and interaction energy for F3330 and AN125, while AN910 showed an increase. MD simulations revealed that the salt ions in the system formed salt bridges between PAM and calcite. Therefore, the adhesion and interaction energy observed from AFM are likely to be the net balance between PAM charged group screening and salt bridging by the salt ions present. Substituting 3% NaCl with equimolar NaNO_3_ in AN910 resulted in higher adhesion and interaction energy in AFM, while NO_3_^−^ ions formed more effective salt bridges than Cl ions in MD analysis. These results confirmed the hypothesis that salt ions with larger bare radii and smaller hydrated radii form better salt bridges.

## Figures and Tables

**Table 1:** AN125 (SPAM) interaction with calcite crystal in 0.1% NaCl.

Polymers	Steric Repulsion Distance (nm)	Batch	Adhesion (pN)		Interaction Energy (aJ)	
			Average	Peak	Average	Peak
AN125	30–70	1	312 ± 130	253 ± 40	24 ± 14	14 ± 3
		2	157 ± 102	83 ± 20	12 ± 9	7 ± 2

**Table 2:** AN910 (10% hydrolysed) interaction with calcite crystal in 0.1% NaCl.

Polymers	Steric Repulsion Distance (nm)	Batch	Adhesion (pN)		Interaction Energy (aJ)	
			Average	Peak	Average	Peak
AN910	40–90	1	41 ± 30	22 ± 4	2.8 ± 2.4	0.09 ± 0.05
		2	55 ± 33	41 ± 4	1.7 ± 1.5	0.12 ± 0.1

**Table 3:** AN125 (25% sulfonated) and AN910 (10% hydrolysed) interaction with calcite crystal in 3% NaCl.

Polymers	Steric Repulsion Distance (nm)	Batch	Adhesion (pN)		Interaction Energy (aJ)	
			Average	Peak	Average	Peak
AN125	50–90	1	169 ± 78	103 ± 20	17 ± 8.1	12 ± 2
		2	71 ± 40	23 ± 10	4 ± 3.7	0.42 ± 0.3
AN910	20–30	1	119 ± 49	89 ± 10	12 ± 6	10 ± 2
		2	120 ± 66	55 ± 10	6 ± 3	3.6 ± 1

**Table 4:** Effect of salt type on the interaction of AN910 (10% HPAM) with calcite surface.

Salt Type	Batch	Adhesion (pN)		Interaction Energy (aJ)	
		Average	Peak	Average	Peak
NaCl	1	119 ± 49	89 ± 10	12 ± 6	10 ± 2
	2	120 ± 66	55 ± 10	6 ± 3	3.6 ± 1
NaNO_3_	1	212 ± 113	183 ± 20	20 ± 11	11 ± 2
	2	167 ± 84	157 ± 20	13 ± 8.7	7.4 ± 2

## Data Availability

Data are contained within the article.

## Supplementary Materials

The following supporting information can be downloaded at: https://www.mdpi.com/article/10.3390/polym16040494/s1.
