# Supplementary material for: Atomic Force Microscopy and Molecular Dynamic Simulation of Adsorption of Polyacrylamide with Different Chemistries onto Calcium Carbonate
Source: Polymers (Basel). 2024 Feb 10;16(4):494. doi: 10.3390/polym16040494 (PMC10893507; doi:10.3390/polym16040494)
Supplement: Supplementary file 1 [file polymers-16-00494-s001.zip › polymers-2835293-supplementary.pdf]

2.2.4. MD simulation methods

Simulation details. All the molecules in the system are modelled with an all-atom forcefield, Polymer Consistent Forcefield-enhanced version (PCFF+), actively developed by MedeA (1) (See (2) for more forcefield parameter details and validation in our previous work), with the forcefield cut-off distance of 9.5Å and treatment of long-range electrostatic interaction with particle-particle-particle Mesh (PPPM) method. System minimization was performed using the conjugate gradient method, and the system was equilibrated under the canonical (NVT) ensemble at 300K for 500ps. This was followed by a production run of of 26ns under the same simulation conditions. All the simulations were conducted with the Velocity Verlet algorithm with a timestep of 1fs. The polymer adsorption behaviour was observed, and the polymer adsorption amount on the calcite was computed. The adsorption surface density is defined as a layer thickness of 4Å region above the calcite surface, where the atoms belong to the polymer chain within this region will be counted and the total adsorbed polymer atom numbers will be normalized by the calcite surface area.

1. Rozanska X, Ungerer P, Leblanc B, Saxe P, Wimmer E. Automatic and Systematic Atomistic Simulations in the MedeA®Software Environment: Application to EU-REACH. . Oil & Gas Science and Technology – Revue d'IFP Energies nouvelles 2014;70 (3):405-17.
2. Hue KY, Lew JH, Myo Thant MM, Matar OK, Luckham PF, Müller EA. Molecular Dynamics Simulation of Polyacrylamide Adsorption on Calcite. Molecules [Internet]. 2023; 28(17).
